# Urine biochemistry assessment in the sequential evaluation of renal function: Time to think outside the box

**DOI:** 10.3389/fmed.2022.912877

**Published:** 2022-07-26

**Authors:** Alexandre T. Maciel, Daniel Vitorio, Eduardo A. Osawa

**Affiliations:** ^1^Research Department, Imed Group, São Paulo, Brazil; ^2^Adult Intensive Care Unit, São Camilo Hospital–Pompéia Unit, São Paulo, Brazil

**Keywords:** acute kidney injury, urine biochemistry, urine, electrolytes, monitoring, urine sodium, fractional excretion of potassium, renal microcirculatory stress

## Abstract

Urine biochemistry (UB) remains a controversial tool in acute kidney injury (AKI) monitoring, being considered to be of limited value both in terms of AKI diagnosis and prognosis. However, many criticisms can be made to the studies that have established the so called “pre-renal paradigm” (used for decades as the essential physiological basis for UB assessment in AKI) as well as to more recent studies suggesting that UB has no utility in daily clinical practice. The aim of this article is to describe our hypothesis on how to interpret simple and widely recognized urine biochemical parameters from a novel perspective, propose the rationale for their sequential assessment and demonstrate their usefulness in AKI monitoring, especially in the critical care setting.

## Introduction

Serum creatinine (sCr) and urine output (UO) have long been used as the cornerstones for renal function monitoring and for the diagnosis of acute kidney injury (AKI). All established AKI criteria (1–3) are based on these two parameters. However, their limitations as real-time biomarkers of renal damage are well-described (4, 5) and sCr is known to increase only after a significant decrease in the glomerular filtration rate (GFR). Changes in creatinine production, volume of distribution as well as tubular creatinine secretion preclude sCr to be an ideal biomarker in GFR fluctuations, a common circumstance encountered by critically ill patients (6, 7). Even though decreases in UO are frequently deemed to be a marker of renal function loss, this is not mandatorily true: oliguria may be a physiological response to states of systemic stress (8).

Urine electrolyte measurement has been proposed many decades ago (9–11) as a useful tool to distinguish AKI caused by low renal perfusion (the so-called pre-renal AKI) from AKI due to structural renal damage, typically attributed to acute tubular necrosis (ATN). The concept of avid Na^+^ retention as a marker of low renal perfusion is still contemporary in most medical textbooks. Albeit these ancient studies have several limitations including insufficient samples, as well as extremely elevated values of mean sCr (inferring advanced AKI at the time of sample collection), low values of both urine sodium (NaU) and fractional excretion of sodium (FeNa) are still interpreted as classic markers of reduced renal blood flow (RBF). Later, fractional excretion of urea (FeUr) was proposed as a better tool to identify pre-renal AKI in the context of diuretic therapy (12, 13) due to the theoretical framework on the minor impact of diuretics on urea reabsorption. Nevertheless, this concept was lately questioned by other investigators (14).

More recent articles have challenged the “pre-renal paradigm,” particularly in septic patients (15, 16). The documentation of low NaU values under normal or even increased RBF seen in hyperdynamic sepsis raised the question of whether urine biochemistry (UB) is useful in the assessment of volemic status and renal perfusion, the main roles played by UB in AKI monitoring so far. The authors then concluded that UB is not a useful diagnostic and prognostic tool in critically ill patients (16). In fact, many articles failed to demonstrate a correlation between urine biochemical values and AKI severity, etiology (17, 18), or current histopathological condition of the kidneys (19, 20). In an attempt to address the paradoxical finding of NaU decrease along with parallel RBF increase described in experimental sepsis (21), we hypothesized that low NaU values could actually be a signal of microcirculatory impairment of the kidneys not mandatorily caused by reduced RBF (22). In fact, dissociation between macro and microcirculation has been frequently reported in septic patients (23). Although their cardiac output may be high, the microcirculatory blood flow is stagnated as pointed out by simultaneous increases (rather than decreases) in sublingual PCO2, another biomarker of microcirculatory impairment (24).

In this article, we aim to suggest simple and feasible alternatives to the classic renal function assessment involving sCr and UO and to show, in our point of view, how sequential assessment of UB may add to real-time, dynamic renal function monitoring.

## Improving the interpretation of serum creatinine and urine output: The role of urine creatinine concentration

Decreases in GFR lead to creatinine excretion impairment with a consequent creatinine accumulation in the blood. Indeed, urinary creatinine excretion is by far the most common pathway for creatinine elimination from the body. Increases in sCr are a late event during AKI development because it is the final step in the process of falling creatinine excretion. Considering a constant creatinine production, sCr rise is preceded by the decline of creatinine filtration, the true marker of a decreasing GFR. Many hours or even days separate these two events: increases in tubular creatinine secretion and body fluid accumulation (25) may delay elevations in sCr. A reliable method for early identification of impairment of creatinine excretion is to quantify the mass of creatinine excreted over a certain time period (6 h, for example). The mass of excreted creatinine is the result of the balance between UO and urine creatinine concentration (CrU), a parameter routinely not assessed in clinical practice (26).

Healthy kidneys have the ability to keep a constant creatinine excretion in a wide range of urinary flows. Therefore, a low CrU does not imply reduced creatinine excretion in patients with high UO. Conversely, prolonged oliguria is common in postoperative patients and not always followed by increases in sCr (27, 28). This is explained by a preserved mass of creatinine excreted per unit of time which means a great ability to concentrate creatinine in a lower urine volume. On the other hand, a disproportional reduction in CrU may compromise creatinine excretion even in the presence of a theoretically adequate or augmented UO: the classic “non-oliguric” AKI, previously called non-oliguric acute renal failure (29, 30). In practical terms, both quantity and “quality” of the urine (31) are equally relevant to renal function evaluation and the “quality” of the urine can be assessed to a large extent by UB.

Although all this seems simple and intuitive, it is important to bear in mind that the interpretation of CrU in the setting of an increased sCr value is more complex. Increases in CrU have been described secondarily to raised sCr values (32). Nevertheless, CrU measurement is useful and must be one of the urine biochemical parameters to be assessed in the dynamic monitoring of renal function, especially in patients with normal sCr values.

## Controversies in the use of fractional excretion of electrolytes in acute kidney injury monitoring

### Are the traditionally measured fractional excretion of sodium and fractional excretion of urea the best options?

It is not feasible to monitor real-time renal function only by using blood parameters, including sCr. The combination of sCr with UO has also several limitations as early markers as described above. Similarly to low NaU and low FeNa (<1%), reduced FeUr (<35%) has been proposed as an indicative of low renal perfusion. However, all of them have failed to be of diagnostic or prognostic value in critically ill patients (16, 33). Again, the main limitation of the studies using FeNa and FeUr is that these parameters were assessed after AKI diagnosis has been made and commonly measured at a single time point. FeNa has an additional limitation: its usual value is already very low in critically ill patients with no AKI (34) so that it is difficult to observe further decline in its measurement in the early phase of AKI development. The advantage of sequential measurements of NaU rests partially in the amplification of FeNa behavior: in other words, a minor reduction in FeNa may lead to a substantial decrease in NaU.

### Fractional excretion of potassium: Should we keep on ignoring it?

We have previously proposed the use of the fractional excretion of potassium (FeK) as a reliable parameter for AKI monitoring (33). In the early course of GFR decrease, FeK increases because there is an augmentation in tubular K^+^ secretion that parallels a decreasing K^+^ filtration in the glomeruli. This phenomenon postpones the rise in serum K^+^ (sK) as well as the decline in K^+^ excretion during AKI development. Both FeK and urinary K^+^ excretion have been proposed as useful parameters in renal function monitoring (33, 35–39). FeK is very different from urinary K^+^ excretion: the former tends to elevate in AKI development while the latter tends to drop. When calculating the fractional excretion of any electrolyte, an elevated result may be directly influenced by increases in sCr. This could be merely a mathematical coupling because sCr is included in fractional excretion’s formula. Thus, a cautious approach is recommended in the interpretation of fractional excretion of electrolytes whenever sCr is augmented. From this point of view, urinary K^+^ excretion has the advantage of not being directly affected by sCr and is promptly available without blood sample collection. It must be obtained over a certain time period, which can be as short as 2 h (36, 37). Although FeK calculation has the practical disadvantage of needing both serum and urinary sample collections, it is usually difficult to interpret urinary values uncoupled with their corresponding blood counterparts.

Considering a normal serum K^+^, FeK formula basically includes sCr, urinary K^+^ concentration (KU) and CrU:


FeK (%)=[(sCr/sK)×(KU/CrU)]×100

Serum K^+^ (sK) and urinary K^+^ (KU) in the same unit (mEq/L or mmol/L) and sCr and CrU also in the same unit (mg/dL or μmol/L).

Once that both sCr and sK are sustained within normal range until the onset of significant decreases in GFR, the (sCr/sK) ratio remains constant in the early stages of renal function loss, at a value around 0.2 to 0.25 (sCr in mg/dL). To obtain this constant, sK and the baseline sCr of the patient are used. For example:


sCr (0.9 mg/dL)/sK (4 mEq/L)->sCr/sK=0.9/4=0.225

Therefore, FeK elevation could be attributed to increases in (KU/CrU) ratio. In normal conditions, this ratio is approximately 0.5 (CrU in mg/dL). A normal range of FeK is then around 10–12% (in the example: 0.5 × 0.225 = 0.1125 = 11.25%) (40).

As a matter of fact, (KU/CrU) ratio is representative of the adequacy of creatinine excretion per unit of urine volume. KU is usually inversely related to UO: oliguric patients have increased KU and polyuric patients have low KU. As mentioned above, because the remarkable signal of creatinine excretion is the mass of excreted creatinine and not its concentration, creatinine can be adequately excreted in either low or high UO. Consequently, oliguric patients may adequately excrete creatinine if they are able to proportionally augment CrU. In other words, if KU elevates in an oliguric state accompanied by a proportional increase in CrU, this implies a preserved ability to excrete creatinine and, in this scenario, oliguria is not followed by a significant sCr increase.

The normal value of KU in a spot urine sample is usually around 40–50 mEq/L (34). For instance, patients with a KU of 40 mEq/L and a CrU of 80 mg/dL are probably excreting a similar mass of creatinine than if they had a KU of 100 mEq/L and a CrU of 200 mg/dL but, in the latter case, a lower UO is expected. In both conditions, the (KU/CrU) ratio of 0.5 in the context of normal sCr argues against subsequent increases in sCr.

In practical terms, FeK may be considered the “future index” of sCr, i.e., it signals the future tendency of sCr. In another example, if a patient has a sCr of 1.0 mg/dL, sK of 4 mEq/L, and a FeK of 20%, subsequent sCr rise is expected, though it does not mean this will mandatorily happen. This situation is particularly common in the immediate postoperative period and it may be an early signal for the risk of AKI development (38, 39). If FeK is 10% instead, elevations in sCr are not expected in the short term. A high (KU/CrU) ratio reflects an imbalance between UO and creatinine excretion in patients with normal sCr levels. Thus, an initial increase in FeK is the result of an increase in (KU/CrU) ratio. Therefore, FeK signals loss of GFR even when sCr and sK have not yet changed. However, in many patients, sCr does not increase postoperatively, even if FeK is high at ICU admission. This is explained by a significant and fast decrease in (KU/CrU) ratio. Such rapid change from a high to a low (KU/CrU) ratio is an interesting physiological observation that corresponds to the swift recovery from the initial renal microcirculatory disturbance induced by the surgery, reducing FeK and precluding increases in sCr. This means that the kidneys in these cases were able to quickly readjust creatinine excretion to the ongoing UO, no matter if urine volume was high or low. Postoperative oliguria may be physiological in patients that have adequate creatinine excretion despite low UO. This is the reason why sCr is not expected to increase in this situation despite the presence of oliguria. Also, it supports the concept that the kidneys are well adapted and no further interventions are needed. Many intensivists would certainly give a fluid challenge based merely on the presence of oliguria, a practice that may cause more harm than benefit. In terms of physiological oliguria, the urine biochemical composition is usually represented by a normal (KU/CrU) ratio due to both high KU (low UO), and proportionally high CrU, suggesting high creatinine concentration in a small urine volume.

Equally important, in a patient with established AKI, the detection of FeK decline may anticipate the identification of resolving AKI as falling measures of (KU/CrU) ratio suggest improved creatinine excretion even before sCr begin to decrease. However, as stated above, the interpretation of urinary biochemical parameters, particularly those involving CrU (all fractional excretion of electrolytes, for instance), represents a difficult task in the background of altered sCr. The advantage of using FeK instead of simply calculating (KU/CrU) ratio is that CrU is normalized to sCr. This is particularly relevant in cases of extreme values of sCr. A malnourished individual may have a high (KU/CrU) ratio due to a low CrU value. However, as the sCr is also very low, FeK remains normal in a steady state.

It is noteworthy that, due to the complex interactions between blood and urinary values included in FeK’s formula, the easiest moment to use FeK is when sCr is close to its baseline value and sK is normal. When sCr begins to increase, the magnitude of subsequent changes in FeK depends on the simultaneous behavior of sK and (KU/CrU) ratio. If this ratio continues to increase, elevations in FeK will be proportionally greater than the rise in sCr alone. Yet, FeK value is unreliable and probably of no utility in severe AKI with very high sCr values. The maximum value FeK can achieve is not determined on physiological grounds and is probably much lower than the value obtained after inputting very high sCr or high (KU/CrU) ratio numbers into the formula.

## The concept of “renal microcirculatory stress”: Anticipating risky situations for the kidneys

Although current AKI criteria are relatively sensitive considering that subtle increases in sCr are diagnostic of AKI, many studies proposed biomarkers of glomerular or tubular injury (41) to evaluate renal damage before function loss *per se*. However, most of these markers are not widely available especially in developing countries and their role in daily AKI monitoring is still not clearly validated. It has been demonstrated that even in a state of early tubular damage as revealed by increases in specific biomarkers, the urine biochemical profile is compatible with “pre-renal” AKI (42). This finding suggests that the decline in NaU occurs very early in AKI development, regardless of simultaneous tubular damage.

### Low urine sodium as a marker of renal microcirculatory stress

**FIGURE 1:**
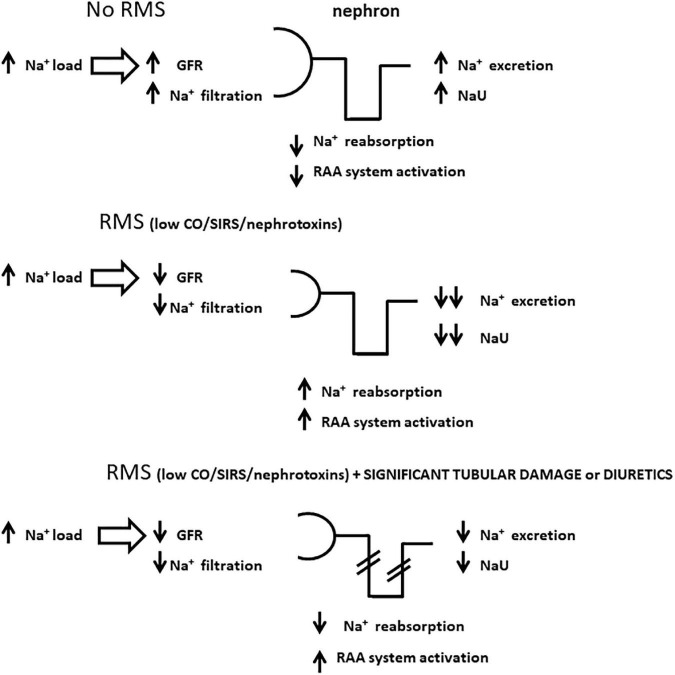
Schematic representations of sodium handling by the nephron in distinct scenarios with high sodium load, a condition frequently seen in critically ill patients. Panel **(A)** represents a state with no renal microcirculatory stress (RMS) where high sodium load generates high urine sodium concentration (NaU), commonly above serum sodium concentration; panel **(B)** illustrates how sodium accumulates in the body in the setting of RMS due to a combination of low sodium filtration and avid sodium tubular reabsorption, which may lead to very low NaU levels; panel **(C)** represents a similar scheme to panel **(B)** except for significant tubular damage and/or the administration of diuretics, both of which jeopardize sodium tubular reabsorption. In this case, NaU levels are also depleted, comparatively higher to panel **(B)**, but not as high as in panel **(A)** due to lower sodium filtration. GFR, glomerular filtration rate; RAA, renin-angiotensin-aldosterone; CO, cardiac output; SIRS, systemic inflammatory response syndrome.

It is known for a long time that the mechanisms responsible for avid Na^+^ retention are located in the macula densa, in close relation to the glomerular apparatus. The activation of both the sympathetic nervous system and the renin-angiotensin-aldosterone (RAA) system has a key role in avid Na^+^ retention. Based on the classic “pre-renal paradigm,” hypovolemia is usually the first diagnostic hypothesis when physicians encounter a low NaU. More recent studies, however, have shown that these Na^+^-retaining mechanisms may be activated under normal or even increased blood flow in renal arteries, as occurs in hyperdynamic sepsis (21). Alterations in glomerular hemodynamics not yet clearly elucidated (43, 44) lead to a reduced glomerular filtration pressure, RAA system activation and, consequently, Na^+^ retention and low NaU levels. In summary, all situations that activate Na^+^ retention may be indicative of conditions causing “renal microcirculatory stress” (RMS) (45) which seems to be a more accurate concept than “pre-renal” AKI (Table 1). Hypovolemia is only one of the wide ranges of etiological factors in the differential diagnosis of RMS. Indeed, we believe that RMS is the common pathway in the early stages of AKI from perhaps all causes, particularly in critically ill patients. Furthermore, even early tubular damage does not prevent from a continuous decline in NaU during AKI progression (42). The reason for this is that tubular damage is not a homogeneous process, and preserved tubules may avidly retain Na^+^. Increases in NaU due to loss of Na^+^-retaining capability is commonly a late and limited phenomenon (34, 42). In this process, Na^+^ filtration, the main source of Na^+^ into the tubules, is concomitantly jeopardized, precluding major increases in NaU (Figure 1). The magnitude of NaU decrease is probably related to the degree of microcirculatory stress and severity of GFR reduction (39). In order to be interpreted correctly, NaU values must be collected sequentially. Only extreme values are relevant on their own: very low values (<20–40 mEq/L) serve as a warning sign and very high values (> 140 mEq/L) infer a favorable state in the critical care setting (see below). We have previously reported a negative correlation between NaU and C-reactive protein (CRP), suggesting a link between NaU and systemic inflammation (46).

**TABLE 1:** Old and new interpretation of urine electrolytes and urinary indices in patients with acute kidney injury (AKI).

	Old interpretation	New interpretation
NaU < 20–40 mEq/L	* Pre-renal AKI * Low renal perfusion –Hypovolemia –Heart failure –Hepatorenal syndrome	* RMS Macrohemodynamic causes: –Low renal perfusion Microhemodynamic causes: –Postoperative SIRS –Sepsis –Trauma –Iodide contrast –Nephrotoxins
NaU 40–140 mEq/L	* Acute tubular necrosis	* Not diagnostic as a single value * Sequential measurements needed
NaU > 140 mEq/L	* Acute tubular necrosis	* Favorable sign * Resolving RMS * Resolving AKI
KU/CrU ratio	* Usually measured to assess hypokalemia: high values suggest renal K^+^ wasting and low values extra-renal K^+^ loss	* Under normal serum K^+^ and sCr levels, high values (>0.5–0.6)^Ω^ suggest an inappropriate creatinine excretion per urine volume
FeK > 10–12%	* Not usually measured in AKI * Previous focus on FeNa and FeUr * Used only to assess dyskalemias	* Risk of AKI development under normal sCr and normal sK levels * “Future index” of sCr * Better accuracy than FeNa and FeUr
FeK < 10–12%	Same as above	* If sCr is increased, it points toward sCr normalization * If sCr is normal, low risk of a sCr increase in the short term

AKI, acute kidney injury; RMS, renal microcirculatory stress; NaU, urine sodium concentration; KU, urine potassium concentration; CrU, urine creatinine concentration; sCr, serum creatinine concentration; FeNa, fractional excretion of sodium; FeUr, fractional excretion of urea; FeK, fractional excretion of potassium.

^Ω^ KU in mEq/L and CrU in mg/dL.

### Very high urine sodium values: Be glad if you find them

In contrast to low NaU, high/very high NaU values (defined here as measurements greater than the value of simultaneous serum sodium) (47) are found in situations where renal microcirculation is not deranged. High NaU values are often seen in patients without organ failure or in those recovering from it, in the absence of significant systemic inflammation (Figure 1). Those patients have usually received a high sodium load in major surgeries or during hemodynamic resuscitation in systemic inflammatory states, including sepsis. A very high NaU results from a rebalance of the total body sodium, of which the excess is excreted by the kidneys (48). Diuretic administration may temporarily lead to NaU augmentation, so its best interpretable value is reached usually some hours (not less than six) after its administration, when the effect is expected to wear off (49). In the context of RMS or AKI, increases in NaU due to diuretic therapy are analogous to increases caused by tubular damage: the elevation is limited because of the simultaneous reduction in GFR, precluding major elevations in NaU to very high values (Figure 1). Of note, the absence of significant elevations in NaU after diuretic administration is associated with a worse outcome (50, 51).

## Urine biochemistry as a tool for an earlier detection of renal dysfunction

In our ICU, we collect a spot urine sample at least on a daily basis in patients who have an indwelling urinary catheter. This sample is collected simultaneously with routine blood samples. In order to minimize urinary catheter manipulation, we collect the spot sample directly from the collecting bag after draining off all urine. We use the first 10–20 ml “fresh” sample of urine (a urine sample that has just been produced by the kidneys) that drains into the emptied bag because it has a greater chance to represent the most reliable UB profile at that specific moment. Both urine and blood samples are sent together for laboratory analysis to enable a proper calculation of FeK.

Urine sodium (NaU) and fractional excretion of potassium (FeK) are certainly more helpful in renal function monitoring when serum urea (sUr), sCr, and sK have normal values. The main objective of measuring those two parameters is to obtain a precise and real-time measure of renal function status prior to sCr increase. Let’s take an example:

A 70-year-old 60-kg male patient was admitted to the ICU with the diagnosis of pneumonia and septic shock. The values of sUr and sCr at ICU admission were 30 and 1.0 mg/dL, respectively, and sK was 4.0 mEq/L. CRP was 20 mg/dL. An indwelling urinary catheter was inserted upon arrival and drained 200 ml of urine. In the spot urine sample, NaU value was 15 mEq/L, KU was 100 mEq/L, and CrU was 120 mg/dL.

**FIGURE 2:**
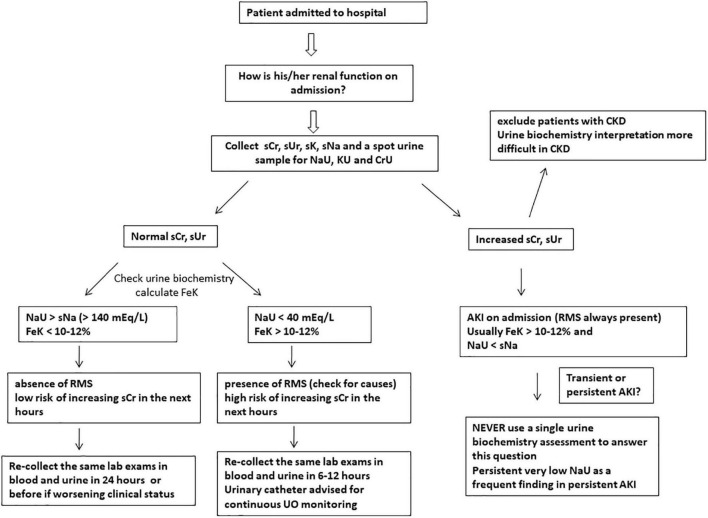
Suggested approach to evaluate renal function at hospital/ICU admission using simple and feasible blood and urinary parameters. Urine sodium concentration (NaU) values between 40 and 140 mEq/L are of uncertain significance when measured at a single time point and should be obtained sequentially. Abrupt decreases in NaU suggest renal microcirculatory stress (RMS) development and risk of acute kidney injury (AKI). SCr, serum creatinine; sUr, serum urea; sNa, serum sodium; sK, serum potassium; KU, spot urine potassium concentration; CrU, spot urine creatinine concentration; FeK, fractional excretion of potassium; UO, urine output; CKD, chronic kidney disease.

**FIGURE 3:**
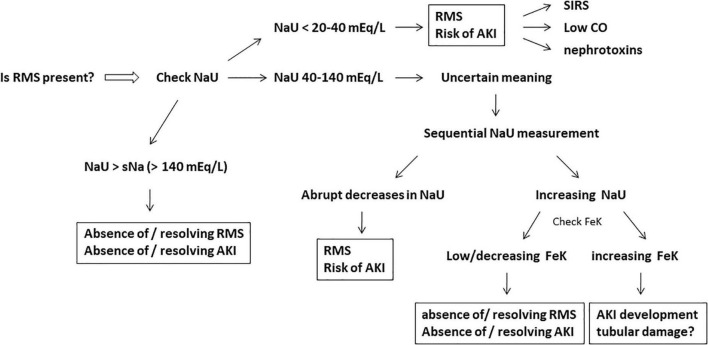
Active investigation for the presence of renal microcirculatory stress (RMS), the initial stage of acute kidney injury (AKI) development. Very low or plummeting urine sodium concentration (NaU) is the hallmark of RMS. The fractional excretion of potassium (FeK) may help to work out the meaning of intermediate values of NaU (40–140 mEq/L) and the subsequent risk of AKI development. Systemic inflammatory response syndrome (SIRS), low cardiac output (CO), and nephrotoxins (iodide contrast/non-steroidal anti-inflammatory drugs/myoglobin, etc.) are potential and frequent causes of RMS. Very high NaU (>140 mEq/L) suggests absence of RMS/AKI or resolving RMS/AKI.

A very low NaU concentration suggests a remarkable RMS. This could be related to either macrohemodynamic factors such as hypotension and hypovolemia, or microcirculatory factors including glomerular circulatory derangements secondary to systemic inflammation/sepsis (Figures 2, 3). Regardless of what caused such an intense Na^+^ retention, it signals an increased risk of AKI. Moreover, it is too early to evaluate UO, because it is unknown how long it took to produce 200 ml of urine. Regarding KU, the high level of 100 mEq/L may serve as a clue as to whether the patient is oliguric at ICU admission. A CrU value of 120 mg/dL results in a (KU/CrU) ratio of 0.83, which is also elevated. Thus, this could be interpreted as a reduced creatinine excretion per volume of urine.

In this case: FeK = (1.0/4.0) × (100/120) = 0.25 × 0.83 = 0.208 = 20.8%

Since FeK is the “future index” of sCr (45), a high FeK (normal value < 10–12%) points toward a subsequent rise in sCr. In fact, FeK normally does increase before sCr in AKI development (33, 38, 52).

The next day, serum and urinary parameters were reassessed: the patient had a 24 h-UO of 500 ml (less than 0.5 ml/kg/h), sCr raised to 1.4 mg/dL, sUr raised to 80 mg/dL, and sK remained 4.0 mEq/L. CRP raised to 30 mg/dL.

Given that sCr is a late marker of renal function, the following greater sCr does not implicate that GFR is still decreasing. It could only be representative of the renal dysfunction which was in progress on the previous day. The new values of NaU, KU, and CrU are, respectively, 8, 80, and 200 mg/dL.

The new FeK value is then: (1.4/4.0) × (80/200) = 0.35 × 0.4 = 0.14 = 14%.

A falling NaU measure is still expected, which emphasizes an ongoing considerable degree of RMS. Such drop in NaU may persist even after hemodynamic optimization, as a result of remaining microcirculatory derangements related to sepsis and persistent activation of RAA system. Decreases in NaU frequently occur in parallel with significant increases in CRP levels, a biomarker routinely linked with the systemic inflammatory state. A falling FeK in the presence of an increased sCr is the result of a decreasing (KU/CrU) ratio suggesting creatinine clearance improvement even in the face of ongoing inflammatory biomarker elevation.

We believe that, in fact, the kidneys were already under recovery on the second day although sCr has increased. This has practical implications: antibiotic dosage, for example, is commonly adjusted for sCr or a calculated creatinine clearance. Should its dose be reduced to suit the newly increased sCr? If we presume that his renal function is recovering, we can then contemplate the maintenance of the dose according to his baseline sCr level.

In our hypothetical patient, in the days that followed successful pneumonia treatment and septic shock resolution, sCr and sUr returned to baseline levels; FeK value gradually decreased to below 12% and NaU reached a value of 155 mEq/L, coinciding with a CRP value of 4 mg/dL. Natriuresis may take longer than diuresis to resolve and, as such, it could be regarded as a more specific marker of inflammatory attenuation. Notwithstanding the identical values of sCr, sK, and sUr at this late time point as compared to the initial measurements, the distinct urine biochemical profile supports that both renal function and microcirculatory status improved markedly during the course of his stay. The progressive decline in FeK implies an incremental excretion of creatinine and a subsequent reduced sCr level. Resolving inflammation along with microcirculatory rearrangement results in improved natriuresis and rising levels of NaU.

Avid proximal tubular reabsorption of sodium is commonly accompanied by avid urea reabsorption (53). Since creatinine clearance improvement and inflammation resolution are not always synchronous, sUr may decrease proportionally less than sCr reflecting a persistent avid tubular reabsorption of sodium and urea. High (sUr/sCr) ratio is frequently associated with “pre-renal” azotemia and hypovolemia (54, 55) but studies failed to correlate such ratio with transient AKI (54, 56). Nevertheless, it seems to correlate with long-term mortality regardless of AKI (57).

Albeit low NaU and high (sUr/sCr) ratio are commonly found together, they are in fact markers of RMS, of which hypovolemia is simply one possible cause. Septic patients may have a low NaU and a high (sUr/sCr) ratio without being hypovolemic. This profile is frequently observed in late stages of AKI, long after fluid resuscitation, as a result of persistent microcirculatory derangements.

## Discussion

Two issues have led to misconceptions over decades regarding UB assessment in AKI. First, UB is usually assessed after increases in sCr have occurred and AKI diagnosis has already been made. The great utility of this tool lies in the period before AKI diagnosis. Second, it is usually measured at a single time point. This practice will not enable a proper interpretation of the measurements because the true value of UB as a diagnostic tool derives from its dynamic behavior over time. Also, the common practice of measuring sCr only once a day certainly contributes to a delayed AKI diagnosis, particularly in situations in which a fast decrease in GFR is expected to be occurring. Nevertheless, urine biochemical changes seem to precede increases in sCr compatible with AKI diagnosis, as was demonstrated by the studies that measured both sCr and UB simultaneously (33, 34, 39).

**FIGURE 4:**
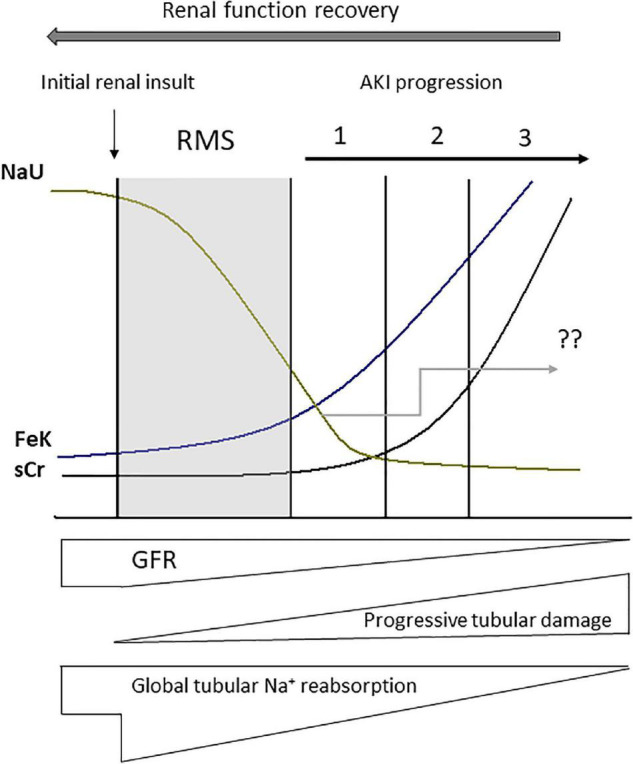
Urine biochemistry behavior after renal microcirculatory stress (RMS) has been triggered by a renal insult (macro or microcirculatory). Abrupt decreases in urine sodium concentration (NaU) as well as increases in the fractional excretion of potassium (FeK) occur before augmentation in serum creatinine (sCr). NaU value after acute kidney injury (AKI) diagnosis is quite variable because it depends on the magnitude of the reduction in glomerular filtration rate (GFR) in combination with the magnitude of tubular damage and impairment of tubular sodium reabsorption, as well as some degree of tubular sodium backleak (“??” represents NaU variability and unpredictability in the Figure). Nonetheless, in the context of AKI, NaU is not expected to reach very high values (>140 mEq/L). These values would only be found after renal function recovery. 1, 2, and 3 correspond to progressive stages of AKI severity.

In most categories of AKI, if not all, critically ill patients will exhibit similar urine biochemical profile in their development phase (34; Figure 4). NaU reduction is commonplace in the context of AKI development regardless of its etiology and duration (34). Moreover, a number of patients with persistent AKI displays long-lasting low NaU values due to persistent RAA activation, tubular sodium backleak and lingering RMS. Such findings, however, may not be observed in patients with advanced and severe AKI (AKI stage three) with a presumed greater degree of tubular damage (34; Figure 4). Old paradigms including AKI with NaU > 40 mEq/L as an attribute of ATN and structural or persistent AKI is questionable: a NaU value of 50 mEq/L observed at an initial time point may plummet to 10 mEq/L in a subsequent measurement.

We have also proposed the use of FeK as the “future index” of sCr. In addition to NaU decrease, which is commonplace in early AKI development, increases in FeK has also been advocated as an early marker of GFR decrease (33). Despite some limitations such as the need for simultaneous blood and urine collection, it seems to be quite more helpful that the traditional FeNa and FEUr values (33, 38). Its major utility is to anticipate changes in sCr and is particularly helpful in patients with normal sCr. Urinary K^+^ excretion is also helpful as it requires only urine sampling and is reliable even when obtained over a period of time as short as 2 h (36, 37). However, it is possible that the early increases in FeK preclude an even earlier reduction in urinary K^+^ excretion. Additional studies are needed to demonstrate whether the dynamic nature of decreasing NaU and increasing FeK value represent a concealed renal microcirculatory stress and a subtle fall in GFR in the context of a normal sCr. It remains to be determined if their routine assessment will enable the guidance of early interventions aimed at modifying AKI course and prognosis.

An additional utility of urine electrolyte measurement is to assist the distinction between physiologic and pathologic oliguria. We hypothesize that the mass of creatinine excreted per unit of time is more relevant than UO alone. Physicians must be mindful that UB monitors renal microcirculation and not specifically renal perfusion. Likewise, oliguria is not a synonym of hypovolemia or low renal perfusion, but may be a signal of RMS.

The behavior of urine electrolytes and indices are similar in early AKI development irrespective of etiology and the magnitude of their changes may, perhaps, be related to subsequent AKI severity and duration. Additional studies are needed to evaluate the sequential UB pattern prior to AKI diagnosis in different scenarios in order to recommend it as a routine monitoring tool in combination with sCr and UO, as well as with glomerular and tubular damage biomarkers. Distinct from other medications that has a theoretical capacity to interfere in urine electrolyte composition (corticosteroids, angiotensin-converting enzyme inhibitors, etc.), diuretic administration is well-documented to interfere both in UO as well as urine composition in a significant way so that their use must always be taken into account during interpretation.

In summary, renal function monitoring must be considered far ahead of the simple monitoring of sCr and UO. It is time to delve into the urinary biochemical composition to expand the renal pathophysiology understanding as long as the values are interpreted properly and in a timely manner. We advocate there is knowledge to be gained by exploring this underestimated and misinterpreted tool. For now, our suggestion is that UB assessment, particularly sequential NaU and FeK measurement should be made in every patient facing a condition of risk to develop AKI while still having a normal sCr. A decreasing NaU and an increasing FeK should alert the intensivist to an ongoing subtle loss of GFR, which may lead to a significant loss of renal function (AKI) if this pattern is not reversed (Figure 4).

## Data availability statement

The original contributions presented in this study are included in the article/supplementary material, further inquiries can be directed to the corresponding author.

## Ethics statement

Ethical review and approval was not required for the study on human participants in accordance with the local legislation and institutional requirements. Written informed consent for participation was not required for this study in accordance with the national legislation and the institutional requirements.

## Author contributions

AM designed this review. All authors contributed to literature revision and manuscript writing and revised and approved the final version of the manuscript.

## Conflict of interest

The authors declare that the research was conducted in the absence of any commercial or financial relationships that could be construed as a potential conflict of interest.

## Publisher’s note

All claims expressed in this article are solely those of the authors and do not necessarily represent those of their affiliated organizations, or those of the publisher, the editors and the reviewers. Any product that may be evaluated in this article, or claim that may be made by its manufacturer, is not guaranteed or endorsed by the publisher.
